# Abuse of and Intervention for Older Adults During the COVID-19 Pandemic

**DOI:** 10.1093/geroni/igab046.329

**Published:** 2021-12-17

**Authors:** Pamela Teaster, Cory Bolkan

## Abstract

Beginning in the United States in 2020, SARS-CoV-2 lead to unprecedented changes in the lives of both younger and older people. Efforts to mitigate the spread of the novel coronavirus, which included physical distancing and self-quarantine not only upended the lives of many people but also created natural laboratory conditions for the mistreatment of older adults. Exploring the mistreatment of older adults during the pandemic presented an unprecedented opportunity to examine perspectives of service providers and affected older adults. This symposium offers four perspectives on this subject. Dr. Karen Roberto and colleagues will present changes and challenges that COVID-19 brought for Adult Protectives Service staff and the vulnerable adults whom they serve. Ms. Lori Smetanka and colleagues will present changes and challenges that COVID-19 created for state and local Long-Term Care Ombudsman. Dr. Holly Ramsey-Klawsnik and Ms. Tammy Seaver will report on how the pandemic affected Nevada Adult Protective Services clients, casework, and staff. Finally, Dr. Pamela Teaster and colleagues will discuss how older adults experienced exploitation attempts during the early months of the pandemic. Dr. Cory Bolkan will begin the discussion, highlighting how conditions brought about by COVID-19 both enabled and thwarted efforts to address elder abuse.

